# A census-weighted, spatially-stratified household sampling strategy for urban malaria epidemiology

**DOI:** 10.1186/1475-2875-7-39

**Published:** 2008-02-29

**Authors:** Jose G Siri, Kim A Lindblade, Daniel H Rosen, Bernard Onyango, John M Vulule, Laurence Slutsker, Mark L Wilson

**Affiliations:** 1 Department of Epidemiology, University of Michigan School of Public Health, Ann Arbor, Michigan, USA; 2 Division of Parasitic Diseases, National Center for Zoonotic, Vector-Borne and Enteric Diseases, Centers for Disease Control and Prevention, Atlanta, GA, USA; 3 Centre for Vector Biology and Control Research, Kenya Medical Research Institute, Kisumu, Kenya

## Abstract

**Background:**

Urban malaria is likely to become increasingly important as a consequence of the growing proportion of Africans living in cities. A novel sampling strategy was developed for urban areas to generate a sample simultaneously representative of population and inhabited environments. Such a strategy should facilitate analysis of important epidemiological relationships in this ecological context.

**Methods:**

Census maps and summary data for Kisumu, Kenya, were used to create a pseudo-sampling frame using the geographic coordinates of census-sampled structures. For every enumeration area (EA) designated as urban by the census (n = 535), a sample of structures equal to one-tenth the number of households was selected. In EAs designated as rural (n = 32), a geographically random sample totalling one-tenth the number of households was selected from a grid of points at 100 m intervals. The selected samples were cross-referenced to a geographic information system, and coordinates transferred to handheld global positioning units. Interviewers found the closest eligible household to the sampling point and interviewed the caregiver of a child aged < 10 years. The demographics of the selected sample were compared with results from the Kenya Demographic and Health Survey to assess sample validity. Results were also compared among urban and rural EAs.

**Results:**

4,336 interviews were completed in 473 of the 567 study area EAs from June 2002 through February 2003. EAs without completed interviews were randomly distributed, and non-response was approximately 2%. Mean distance from the assigned sampling point to the completed interview was 74.6 m, and was significantly less in urban than rural EAs, even when controlling for number of households. The selected sample had significantly more children and females of childbearing age than the general population, and fewer older individuals.

**Conclusion:**

This method selected a sample that was simultaneously population-representative and inclusive of important environmental variation. The use of a pseudo-sampling frame and pre-programmed handheld GPS units is more efficient and may yield a more complete sample than traditional methods, and is less expensive than complete population enumeration.

## Background

Malaria remains a critical health problem in sub-Saharan Africa (SSA), with about 1 million deaths [1] and 365 million cases each year [2]. Several factors have led to a growing recognition of the specific importance of urban malaria in SSA. While most Africans still live in rural areas, the proportion of urban dwellers in SSA is significant (35.2% in 2005), and growing at a rate close to double the world average (3.6% versus 2.0%/year from 2000–2005), such that the proportion of Africa's population living in urban areas will exceed 50% by 2030 [3]. Although malaria continues to be a primarily rural disease, a recent review indicated that 6–28% of global malaria cases might arise in urban settings in SSA [4].

Demographic, ecological and behavioural factors suggest that the epidemiology of urban malaria is likely to be very different from that in rural areas [4-6]. Studies attempting to characterize risk factors for urban malaria face many challenges in sampling design, including the lack of pre-existing sampling frames, heterogeneity in the distribution of populations, micro-environmental variation, and the spatially focal nature of malaria transmission in cities. In response to these obstacles, researchers have adopted various strategies, including *convenience samples *from health facilities [7-13]).) or schools [9,11,14]. *complete enumeration *of residents within a limited study area [15-18] and *cluster sampling *from population or geographic strata – which may or may not involve complete enumeration [19-22]. A few studies have used census data, but such data are rarely available and population turnover in low-income areas limits their utility [23]. These efforts are usually tailored to answer specific questions; for example, the World Health Organization (WHO) rapid urban malaria appraisal (RUMA) methodology uses school and health facility samples to establish an overview of the malaria situation within a defined urban area prior to more detailed research and/or control initiatives [11].

Any sampling strategy involves tradeoffs between population-representation and extent of coverage, and must also take into account existing data and available research resources. Convenience samples, which are limited by the catchment areas of the data source in physical and sociological space, may fail to accurately represent either the urban environment or the overall population. For example, a sample from a hospital can only account for the proportion of the population within a specific geographic area and range of socio-economic status that would attend that hospital in a health emergency. This sample may or may not represent the overall population, and limits study of environmental risk factors to ecotypes observed within that specific area. Complete enumeration is costly, thus limiting the size of the area that can be sampled, whether as a cohesive geographic unit or as a series of clusters within a larger urban area. Cluster sampling is further limited by design effects resulting from high intra-cluster correlation of variables. Where clusters are chosen on a geographic basis, or where sampling is geographically random, over-sampling of sparsely-populated areas that cover a proportionately larger fraction of the study area is a likely result. Moreover, the focal nature of malaria transmission in urban environments, partly due to limited vector mosquito dispersal [14,24]. implies that cluster sampling strategies which fail to include significant urban ecotypes may underestimate the importance of urban malaria and/or produce inaccurate estimates of risk factor effects.

Questions of efficiency also limit the scope of population-based sampling strategies. Indeed, the location and identification of specific individuals in cities requires interviewers with considerable knowledge of the sampling area, and may entail an effort that offsets the relative gains in efficiency brought about by physical proximity to governmental and research institutions. Furthermore, the high mobility and cultural and social diversity of city dwellers requires substantial training and flexibility on the part of interviewers. Population turnover is extremely high, especially in low-income areas, and can lead to attrition in the selected sample.

While most of these issues are not unique to urban areas, the absolute size of populations and the diversity of epidemiological and environmental contexts are generally greater than in rural areas of comparable geographic size, making the design and implementation of sampling a more complex problem. Accordingly, a new sampling method was used to characterize the malaria-endemic urban region of Kisumu, western Kenya. In particular, a spatially stratified household sample was constructed to jointly evaluate environmental and socio-demographic factors as they varied across this large urban area. The sampling strategy was designed to simultaneously provide population-representativeness, comprehensive coverage of inhabited ecotypes, and reasonable cost-efficiency. This article describes the application of this novel strategy to select a representative sample for a knowledge, attitudes and practices (KAP) survey of malaria. The current method approximates a true population-based sample, while eliminating many of the costs of identifying and interviewing specific sampling units.

## Methods

### Study site and malaria patterns

Kisumu, Kenya (pop. 326,407; 1999 census), on the shores of Lake Victoria, is the third-largest urban area in the country [25]. Its size makes it representative of the setting in which most SSA population growth will occur over the next 30 years[3]. In order to select an "urban" study area, data collection was limited to the 13 administrative sublocations of the city (roughly equivalent to large neighbourhoods) with overall population densities > 1,000/km^2^. This density threshold has been used to define "urban" in a recent review of malaria morbidity and mortality across Africa [26]. At smaller scales, population density within the study area varied considerably. The study area encompassed a range of urban ecotypes with variation in factors likely to influence malaria risk, including land use/land cover, economic and agricultural activity, distance from urban shops and health facilities, and environmental features. The study site comprised 202,282 people in 54,403 households (Table 1) [25], over an area of 62.3 km^2^.

**Table 1:** Summary of geographic units and people for Kisumu and study area. Units and percentages are displayed for the city of Kisumu and the study area, along with the intended and actual study samples.

**Unit**	**Total in Kisumu**	**No. in Study Area**	**No. in Intended Sample (% of study area)**	**No in Actual Sample (% of Study Area, % of Intended Sample)**
Sublocations	36	13	13 (100%)	13 (100%, 100%)
Enumeration Areas	788	567 (535 "urban" and "32 rural")	567 (100%)	473 (83.3%, 83.3%)
Households	82099	54403	5479 (10.1%)	4336 (8.0%, 79.1%)
People	326407	202282	n.a.(n.a.)	20797 (10.3%, n.a.)

Malaria incidence in rural areas surrounding Kisumu, mostly due to *Plasmodium falciparum *[27], is among the highest in East Africa [27,28]. Transmission is greatest following the two rainy seasons that typically occur during April-June and October-December. Malaria is consistently the leading reported cause of outpatient and inpatient morbidity and mortality among children [29].

### Sampling strategy

**Figure 1:**
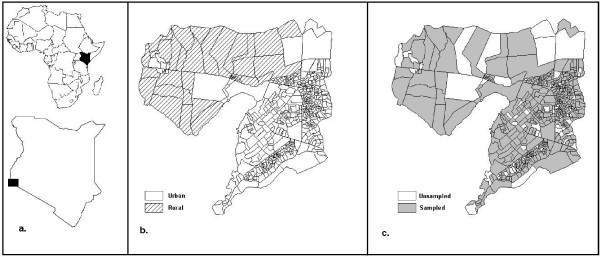
**Maps of study area in Kisumu, Kenya**. Maps include (a) region of study, (b) urban/rural designation of census enumeration areas (EAs) within study area, and (c) sampled and unsampled EAs.

The sample was designed to select 1-in-10 households citywide, stratified by census enumeration area (EA), as defined by the Kenya Central Bureau of Statistics during the decennial national census. Census guidelines specify that each EA should ideally comprise ~100 households, but this varied where population density or environmental features required larger or smaller boundaries to facilitate enumeration [25]. EAs are designated by the census as either "urban" or "rural." Each EA represents a geographic stratum in this stratified sampling design, which was comprehensive, including all EAs within the study area. Summary EA-level population data and detailed sublocation maps were obtained from the Kenya Central Bureau of Statistics and used to construct a pseudo-sampling frame, i.e., a list of census-sampled structures representing the geographic distribution of the population. Sublocation maps indicated EA boundaries and sampled structures for all EAs designated as urban by the census (n = 535; Figure 1), while census data provided the number of households. Using this information, a sample of structures was randomly selected equal to one-tenth the number of households in the EA (Table 1). For EAs within the study area that were designated as rural by the census (n = 32; Figure 1), census maps did not display structures, so a geographically random sample equal to one-tenth the number of EA households was chosen from a grid of potential sampling points regularly spaced at 100 m intervals.

Locations of sampled structures, for urban EAs, or random sampling points, for rural, were noted on the sublocation maps, and cross-referenced to a geographic information system (GIS) map of the study area developed by the US Centers for Disease Control and Prevention (CDC) in conjunction with the Kenya Medical Research Institute (KEMRI) (Ombok M, *pers. comm*.). Each identified location represented a sampling point for the survey. Trained interviewers transcribed the coordinates for each sampling point from the GIS into a handheld Garmin ETrex Global Positioning System (GPS) unit (Garmin, Olathe, KS). These interviewers used the GPS unit to locate the sampling point, applying a standard protocol to identify the closest household with a resident child under 10 years old, and attempting to interview the primary caregiver for that child.

### Interviewer training, interview protocol and sample validity

Interviewers underwent six weeks of training in quantitative interviewing techniques, mapping and GPS use. They were tested on their ability to locate specific points prior to the start of data collection. Standardized procedures were established to identify the closest eligible house to an assigned sampling point, as were procedures for selecting specific households or children within households where more than one was eligible.

Each interviewer was assigned a random sample of EAs to minimize interviewer bias. The order in which EAs were approached was also randomised, and all interviews for a specific EA were collected consecutively for logistical reasons. Interviews were conducted during daytime hours, except where the respondent's schedule dictated otherwise. Interviewers travelled to the assigned EA in the morning with a list of sampling point coordinates for that EA. Upon navigating with the GPS unit to the first sampling point, they examined the nearest structure and determined whether it was residential or other (e.g., commercial or industrial). If clearly non-residential, the interviewer proceeded in a clockwise spiral outward from the sampling point, until a residential structure was identified. Where neighbourhood or street configuration required a different approach, interviewers made an attempt to choose the closest available residence to the sampling point within those constraints. Where a structure had ambiguous function, or was clearly residential, but no one was home, the structure was GPS marked, and the interviewer proceeded to the next sampling point before returning at a later time.

Once a residence was identified, the interviewer ascertained whether the household had an eligible child, and if so, whether the primary caregiver was present. When the caregiver was absent, three attempts, at different times of day, were made to complete an interview before moving to the next available household. If more than one child in a household was eligible as the subject of an interview, one was selected based on a prearranged random number. Random selection was also used when more than one household was available in a single-structure. If a single structure contained > 10 households, extra interviews were conducted to maintain the 1-in-10 sample protocol. A supervisor occasionally accompanied interviewers to monitor equivalence of technique and provide feedback to the primary investigator, and separately re-interviewed a subset of households to ensure data completion and assess accuracy. In sparsely populated or semi-rural areas, a representative of the local sublocation familiar with the locations of residences occasionally accompanied interviewers as a guide. The interviewers, supervisor, and primary investigator met weekly or more frequently as needed, to discuss interviewing problems and review upcoming interview assignments.

Sample validity was assessed by quantifying completion and non-response rates, and by comparing the basic demographics of the selected sample with those reported by the 1998 Demographic and Health Survey (DHS) [30]. Sampled EAs were compared to non-sampled EAs on variables of interest, where possible. Accuracy of sampled points was estimated by comparing positions of sampled interviews to the original sampling point coordinates and determining whether interviews were completed within the assigned EA. For all analyses, the sample selected via the map-based strategy for administratively urban EAs was compared with that selected through a random geographic process for rural EAs.

### Human subjects

The protocol for this study was approved by the Kenya Medical Research Institute (KEMRI) National Ethical Review Committee (Nairobi, Kenya) and the Institutional Review Boards for the CDC (Atlanta, GA) and the University of Michigan (Ann Arbor, MI). The research protocol and rights and responsibilities of participants were explained to potential respondents by interviewers, and written informed consent was obtained prior to all interviews.

## Results

### Sample completion

Of 567 EAs in the study area, 473 (83.3%) were sampled from June 2002 through February 2003, yielding 4,336 valid interviews. Although data collection was curtailed for logistical reasons, non-sampled EAs were randomly distributed (Figure 1). There was no difference between the sampled proportions of urban (83.6%) and rural EAs (84.4%; χ^2 ^= 0.015, p = 0.90), or between the mean populations of sampled (353.4) and non-sampled EAs (374.1; t-test, p = 0.27).

Of the 4,551 assigned sampling points, nearly all (4,336 or 95.3%) yielded an interview. Non-response and refusal were minimal: in 357 cases (7.8% of assigned sampling points), more than one household had to be approached to obtain an interview. Of these, 270 households (75.6%) were ineligible because there was no child under 10 years old. In 55 cases (15.4%), no caregiver could be contacted, despite the presence of an eligible child. In 39 otherwise eligible households (10.9%), all caregivers refused to participate. The overall non-response rate (i.e., the proportion of identified eligible households where interviews were not completed because no caregiver could be contacted or all caregivers refused) was just 2%.

### Distance to sampling points

Because the sampling procedure did not specify a particular individual or household for interview, distance from the assigned sampling point to the sampled household varied. Mean distance to sampling point was 74.6 m, significantly less when using sampling points based on census-sampled structures in urban EAs (66.6 m; 95% CI: 57.6–75.6 m) than when using geographically random sampling points in rural EAs (158.6 m; 95% CI: 131.0–186.1 m; t = 5.95, p < 0.0001). This difference remained significant after adjusting for the number of households in the EA (t = 2.22, p < 0.03). Since distances to assigned sampling points varied, some interviews were eventually performed in EAs other than that originally designated. Thus, although 473 EAs were initially assigned sampling points, interviews were eventually conducted in 511. The final mean sampling fraction of households per EA was 9.1%, slightly less than the planned 10%. Overall, 2,398 (55.3%) interviews were performed in the assigned EA, with no significant difference between proportions sampled in EAs designated as urban (55.0%) versus rural (57.9%; χ^2 ^= 1.20, p = 0.27).

### Demographic characteristics

**Figure 2:**
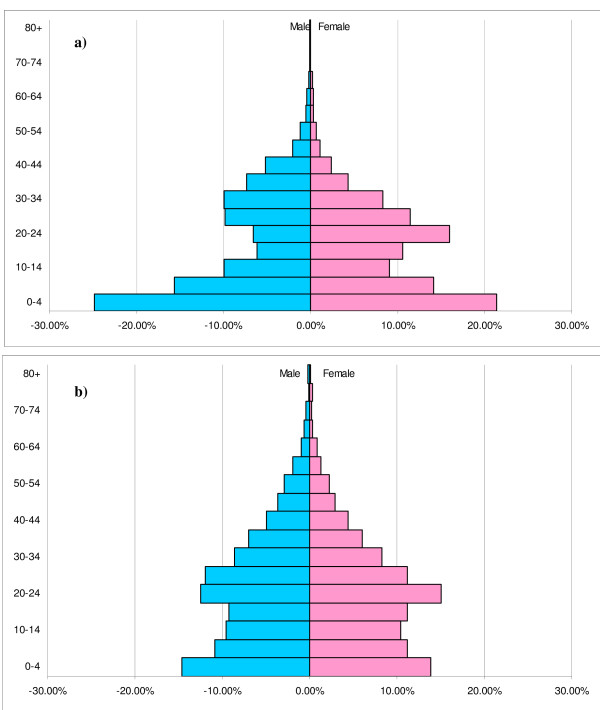
**Comparison of population age structure in study area with urban Kenya**. Population age pyramids are shown for (a) study area and (b) urban Kenya. Data for urban Kenya is reproduced from the Kenya Demographic and Health Survey [30].

The population distribution by age and sex for the sample was compared to that obtained from the 1998 Kenya DHS (Figure 2). Most age and sex groups were not statistically different (data not shown). The biggest discrepancy was a significantly higher proportion of the study sample in the youngest two age groups (i.e., < 5 and 5–10 years) than observed in the DHS (38.0% versus 24.9%; χ^2 ^= 384.5, p < 0.0001). Also, males aged between 15 and 30 years were significantly underrepresented in the study sample (10.8%) compared to the DHS (16.5%; χ^2 ^= 118.0, p < 0.0001), as were females over 35 (4.9% versus 8.5%; χ^2 ^= 178.6, p < 0.0001), and males over 45 (1.2% versus 5.7%; χ^2 ^= 397.2, p < 0.0001).

## Discussion

Although malaria in urban environments has become increasingly important, sampling methods used in rural settings may not, in some cases, effectively address the special circumstances of urban conditions. This project applied and tested a method for identifying population-representative samples of people in complex urban settings that may have different malaria risk parameters from rural sites. Given the population and environmental heterogeneity of urban environments, the focal nature of urban malaria transmission, and the lack of pre-existing sampling frames, the goal was to develop a population-representative sampling approach that simultaneously accounted for environmental variation, while not requiring strict enumeration. Such an approach should be both accurate with respect to the population parameters measured and sufficiently inexpensive and straightforward for adoption by local health ministries and researchers.

### Age- and population-specific representation

The sampling strategy was designed to select a sample representative of the target population of households with children aged < 10 years in the study area. The age profile of the sample population varied in predictable ways from the DHS data collected for urban areas in Kenya (Figure 2). Since only households with children < 10 were eligible, there was an excess of children in the lowest age groups, and a relative dearth of individuals beyond child-bearing age, relative to the general population. The under-representation of adolescent and young-adult males probably indicates that males in this age range are less likely to be caregivers of young children, or are more likely to live in households of their own. Since DHS and government census household data have been presented as summaries (i.e., not separately for households with young children), quantitative appraisal of the accuracy of this sampling strategy in capturing the true target population is difficult. Moreover, as detailed population data were not available for Kisumu itself, the conclusions presented here may be inaccurate to the extent that the urban DHS data misrepresented this population. However, the observed age distribution was consistent with the expected age structure of a representative sample.

Other considerations suggest that the method identified a sample representative of the target population. While the sample was not based on a true, fully enumerated sampling frame, it made use of enumeration-based aggregate census data over small spatial units. Recent census maps (1999) were employed, such that spatial units and populations had probably changed very little by the time this study was conducted (2002). Indeed, a GIS-based comparison of census maps with aerial photographs of Kisumu from 1996 and high-resolution satellite images from 2003 showed few differences, indicating that the former accurately portrayed the geographic locations of buildings, whether or not inhabited at the time of the current investigation. Inhabited structures may not be represented on maps where rapid urbanization or substantial migration of residents is occurring, or where the time between map creation and sampling is lengthy. However, since individual residential changes generally occur at a more rapid rate than regional urbanization, using sampling points that reflect the overall geographic distribution of population rather than selecting individuals from a pre-existing fully enumerated sampling frame should increase efficiency and accuracy, as the sample will not be missing persons or households that are no longer present.

### Household-based strategy and implementation

In addition to sample design issues, the manner in which sampling is implemented may affect representativeness. In this study, the proportion of houses that was recorded as ineligible because no eligible child was present was less than 6% of all attempted interviews. This is lower than expected, since 11.3% of households in Nyanza province, where Kisumu is situated, are single-member [30], and some multi-member households also lack eligible children. It is possible that Kisumu city differs in this respect from the overall estimate for Nyanza province. Otherwise, interviewers may have unconsciously tended to approach households that were more likely to have eligible children or interviewers, despite being trained and tested in the use of standardized algorithms for selecting the household closest to the sampling point, or failed to record unsuccessful sampling efforts. Most likely, a combination of these factors produced more than expected eligible households. Regardless, this should not produce biased results unless interviewers selected households that were more likely to agree to participate. Information from declining households was not extensively evaluated, but the lack of apparent spatial pattern among them suggests that the implementation of the sample did not affect its representativeness.

### Administrative units and sample design

Data collection was curtailed after 83% of the total EAs were sampled, for logistical reasons and because a sufficient sample had been attained for the purposes of the research. This could potentially have affected either the internal or external validity of the sample. Because sampling did not occur in certain EAs, estimates of population parameters for these areas were not possible. However, no observable differences between sampled and non-sampled EAs were found, suggesting that the lack of complete coverage did not produce sampling bias. Indeed, the substantially broad geographic coverage of the study area suggests that the sample retained internal validity. Moreover, the observed non-systematic distribution of non-sampled EAs (Figure 1) and the field team's on-the-ground knowledge of the study area also suggest that no significant urban ecotypes were omitted.

### Geographic error and sampling error

There were geographic discrepancies between some actual interview locations and the originally assigned coordinates for sampling points, which likely arose from several sources. GPS measurements contain inherent error (~5–15 m) that introduces uncertainty to the observed locations of interviewed households. Failure to find an eligible household at the sampling point and vagaries of local neighbourhood structure also may have forced interviewers to travel further in search of an eligible house. If this variability created under- or over-sampling in particular geographic strata, the accuracy or precision of parameter estimates in those areas could have been affected. Misclassification error (e.g. interviews conducted in an EA other than the assigned EA) apparently was greater in poor urban and peri-urban areas, perhaps due to the small geographic size of EAs in these areas. Nevertheless, the census map-based sampling strategy used in urban EAs yielded significantly shorter distances between assigned and sampled points than did the geographically-random sampling used in rural EAs, indicating a correspondence between the actual distribution of population and the distribution of sampling points generated by the sampling process. While this method does not have the accuracy obtained by a population-based sample at finer scales with complete enumeration, aggregation of EAs into larger areas for analysis removes the positional and demographic uncertainty associated with geographic deviations, thereby improving the precision of parameter estimates for those areas.

In addition to sampling-based geographic error, inaccuracies in the census maps were observed, particularly in poor urban and peri-urban areas. In general, if such inaccuracies lead to errors in mapping EA boundaries to the GIS and ground coordinates, the accuracy of parameter estimates will be diminished, potentially biasing the sample if the "true" EA encompasses a larger or smaller population than that identified by the map. However, this type of misclassification also should be non-differential, and diminishing with aggregation of EAs for analysis.

### Comparison with other sampling methods

The sampling design presented here has several benefits over traditional sampling methods. First, the use of a stratified sample both eliminates the design effects associated with cluster sampling and should increase the precision of survey estimates across the study area compared with a random sample. Second, the use of fine-scale geographic stratification at the EA level constrains the sample to include all inhabited environments within the study area, not only lessening the likelihood of missing important variation by chance, but also ensuring near-continuous measurement of environmental variables across the city, thus allowing for better spatial modelling of variation. Third, the use of positional data from census maps increases the population-representativeness of the sample, since it mirrors population distribution on the ground better than a random geographic sample, as is demonstrated here, and significantly better than a convenience sample from, for example, a hospital or school.

Cost and efficiency represent important benchmarks beyond simple accuracy and validity for any sampling strategy to be applied in SSA. The current approach included several features that, for urban areas, may represent improvements over traditional sampling methods, and be of particular use where rapid sampling is needed. Although this approach required sampling from widely dispersed geographic strata, increasing costs compared to those expected using a highly clustered sampling strategy, the use of pre-programmed handheld GPS units and positional population data from census maps partially offset this, decreasing interviewer training costs and interviewing time, as interviewers were guided to the appropriate sampling point, even without prior knowledge of the study area. Retraining costs for future studies are also obviated in part, as interviewers do not need to be familiarized with new areas or study sites to nearly the extent necessary if particular individuals or households must be identified. Most importantly, the costs of complete enumeration within clusters are avoided if reasonably accurate summary population figures are available, as was the case here. This study made use of both pre-existing population summary figures and census maps, elements that normally constitute a large proportion of the expenditures of a population enumeration [31]. Similar data should be available in some format for many SSA cities, and other types of data (e.g., land tenancy maps or remote sensing images) may be adapted for identifying map-based sampling points. A strict analysis of cost-efficiency was not performed for this method in comparison with traditional methods, in part because some benefits are essentially unquantifiable in the context of a single project (e.g., the relative decrease in retraining costs), but also because the costs incurred by the national census to produce the summary data used here were unavailable, and because the implementation of a cluster sample of comparable extent was beyond the logistical scope of this project. However, such a comparison would be worthwhile in the context of urban malaria research.

Another sampling strategy for malaria epidemiology in urban areas has been developed for Kisumu. Keating et al. [32] describe a geographic sampling strategy for ecologic studies, focusing on relationships between anopheline larval ecology and human activity. Briefly, the study area, encompassing an urban zone broadly similar to that in this study, was subdivided into a grid of 270 m × 270 m squares. This grid was stratified by drainage (well vs. poorly-drained) and level of planning, and proportionate sampling from the four strata was used to select cells for further sampling. Approximately 100 interviews were completed for each stratum.

Compared with this geographic sampling strategy, the current method is likely to offer greater population-representativeness, since the geographic sampling unit (EA) is structured on population density and household distribution, and because the pseudo-sampling frame developed here mirrors the presence of inhabited structures on the ground. This is especially true where a grid cell, by chance, encompasses a sparsely populated area. In addition, the prior method was designed in part to evaluate the effect of two important variables on anopheline ecology. In stratifying by these variables, it is likely better able to resolve their effects than is the current study. However, because sampling does not necessarily reflect intra-urban population distribution, it is probable that some inhabited microenvironments are undersampled with respect to the proportion of the populace they affect, making the resolution of other environmental risk factors less reliable than with the current method, where coverage of inhabited microenvironments is comprehensive. Finally, the sample selected by the current project is substantially larger, allowing for an increased ability to evaluate intra-urban risk factors, or to examine risk factors for smaller regions within the city, as well as to model spatial variation in risk factors or outcomes more continuously across the study space. While the earlier project aimed rather to infer characteristics of the study area using a small, well-selected sample than to sample comprehensively, it is noteworthy that the larger scheme described here resulted from the lack of reliance on complete enumeration of households and the use of GPS to guide interviewers, which made it feasible to sample all EAs, rather than a small fraction. It is important to note that these two strategies reflect substantially different design priorities and research goals, which they are separately effective at addressing.

It is noteworthy that designation of the study area as "urban" and identification of appropriate boundaries was not straightforward. There is no standard definition for urban in SSA. National guidelines variously draw on population density thresholds, absolute population sizes, proportions of residents in various occupations and other functional characteristics, or, tautologically, on administrative boundaries [26]. In this study, population density was used to define the limits of the study area, as the most widely available and generally applicable of these measures, while recognizing that a suite of other factors are jointly and interactively responsible for determining what is "urban." A recent review of urbanization and malaria used the threshold of 1,000/km^2 ^to define "urban" in estimating malaria morbidity and mortality across Africa [26]. Clearly, different valuations of the variables used to define urbanicity would have led to different study areas and overall parameter estimates for the current study. However, in the absence of a standard definition, equally valid studies of the same city are likely to yield different and irreconcilable parameter estimates. The same holds true for intra-urban classification schemes that use terms such as peri-urban, semi-urban or even suburban without specifying what is meant by these terms. Further research is needed to identify the salient characteristics of urban versus rural malaria, and the factors that should determine urban boundaries in the context of malaria research.

The utility of the sampling strategy introduced here is not limited to KAP surveys, but should be applicable to any study that requires a population-representative sample across a large urban area, particularly where environmental and socio-demographic factors are being jointly evaluated. In conjunction with serological or entomological work, it would represent a valuable intermediate step in establishing the malaria status of a city, more time-consuming than a convenience sample, but more detailed and accurate, with the added benefit of identifying specific areas of high-risk, since the entire inhabited environment is sampled. It should be of particular use in research that models continuous spatial variation of malaria risk or of environmental or behavioural variables across an urban landscape, or where risk parameters for multiple neighbourhoods within a city are to be evaluated.

## Conclusion

This new, census-weighted, spatially-stratified sampling strategy successfully identified households in Kisumu, Kenya that were simultaneously representative of the population at risk for malaria in this urban environment of SSA and of the urban environments where people live. This strategy, based on population counts and maps from a recent census, and handheld GPS to identify sampling points selected from these maps, should be useful in other similar settings. It addresses many of the sampling difficulties in urban settings of SSA, and may offer improvements in terms of representativeness and cost-efficiency over traditional sampling methods for such urban areas.

## Competing interests

The author(s) declare that they have no competing interests.

## Authors' contributions

JGS was primarily responsible for the conception and field implementation of the sampling strategy, the statistical analysis of results and the production of the manuscript draft and revisions. KAL participated extensively in study design, supervised the field project, and contributed to the statistical analysis and critical review and revision of the manuscript. BO coordinated fieldwork and provided practical input on sample implementation. DHR contributed to study design and managed data processing and verification. JMV and LS played supervisory roles. MLW provided critical input on study design, statistical analysis and revision of the manuscript. All authors read and approved the final manuscript.
